# Preparation and Characterization of In Situ (TiC-TiB_2_)/Al-Cu-Mg-Si Composites with High Strength and Wear Resistance

**DOI:** 10.3390/ma15248750

**Published:** 2022-12-08

**Authors:** Yu-Yang Gao, Ying Liu, Yuan-Lin Li, Ang Zhang, Hang Teng, Zhi-Hua Dong, Tian Li, Bin Jiang

**Affiliations:** 1 College of Materials Science and Engineering, Chongqing University, Chongqing 400044, China; 2 National Engineering Research Center for Magnesium Alloys, Chongqing University, Chongqing 400044, China; 3 Nanjing Yunhai Special Metal Co., Ltd., Nanjing 211212, China; 4 Grainger and Worrall Ltd., Bridgnorth WV15 5HP, UK

**Keywords:** in situ multi-sized TiC-TiB_2_, Al-Cu-Mg-Si composites, abrasive wear properties

## Abstract

This study involved the preparation and characterization of in situ (TiC-TiB_2_)/Al-4.7Cu-0.32Mg-0.44Si composites with excellent mechanical and abrasive wear properties. The composites were synthesized in an Al-Ti-B_4_C system by combining combustion reaction synthesis with hot-pressed sintering and hot extrusion. The in situ TiB_2_ and TiC particles were of multi-scaled sizes ranging from 20 nm to 1.3 μm. The TiB_2_ and TiC particles effectively increased the yield strength (*σ*_0.2_), ultimate tensile strength (*σ*_UTS_), hardness (HV), and abrasive wear resistance of the composites. The 40 wt.% (TiC-TiB_2_)/Al-4.7Cu-0.32Mg-0.44Si composite exhibited the highest *σ*_0.2_ (569 MPa), *σ*_UTS_ (704 MPa) and hardness (286 HV), which were 74%, 51% and 110% higher than those of the matrix alloy, respectively. Compared with the matrix alloy, the abrasive wear resistance of the 40 wt.% (TiC-TiB_2_)/Al-4.7Cu-0.32Mg-0.44Si composite was increased by 4.17 times under an applied load of 5 N and Al_2_O_3_ abrasive particle size of 13 µm. Micro-ploughing and micro-cutting were the main abrasive wear mechanisms for the Al-Cu-Mg-Si alloy and the composites.

## 1. Introduction

Al matrix composites possess great potential as lightweight critical components in the aero and automotive industries because of their excellent specific strength, specific modulus, and superior wear resistance and fatigue behaviors [1,2,3,4,5,6,7]. Demand for lightweight, high-strength materials has prompted scientific researchers to develop Al matrix composites with enhanced mechanical properties and improved wear resistance [8,9,10,11,12,13,14,15].

Micron-sized ceramic particles in Al matrix composites have been reported to increase the strength and hardness of the composites [16,17]. Ceramic particles also effectively resist the penetration of abrasives and reduce the abrasive wear of the composites [18,19]. However, the strength and plasticity of Al matrix composites reinforced by nano-sized ceramic particles are higher than for composites reinforced by micron-sized particles [20]. Nano-sized ceramic particles have been shown to significantly increase the plastic deformation resistance of the composites [6,21]. Based on our previous investigations, Al matrix composites reinforced with bi-modal-sized ceramic particles showed an improved distribution of ceramic particles and exhibited excellent elevated temperature mechanical properties compared to composites reinforced with single-sized particles [22,23]. Zhang et al. reported that the *σ*_0.2_ (358 MPa) and *σ*_UTS_ (585 MPa) for hybrid-sized SiC/Al-Cu matrix composite (40 nm + 15 µm) were higher than those for single nano-sized SiC/Al-Cu composite (312 MPa, 512 MPa) and single micron-sized SiC/Al-Cu (302 MPa, 503 MPa) composite [22]. Dual-phase-ceramic-particle-reinforced Al matrix composites have attracted the attention of researchers due to their enhanced mechanical properties and wear resistance [23,24,25,26]. As reported by Gao et al., dual-phased (TiB_2_-Mg_2_Si)/Al composite showed more uniformly distributed particles and higher strength than single-phased TiB_2_/Al and Mg_2_Si/Al composites [26]. The *σ*_0.2_ and *σ*_UTS_ of dual-phased (TiB_2_-Mg_2_Si)/Al, single-phased TiB_2_/Al, and single-phased Mg_2_Si/Al composites were 141 MPa and 217 MPa, 74 MPa and 147 MPa, and 116 MPa and 201 MPa, respectively. Kumar et al. compared the tribological properties of Al matrix composites reinforced by dual-phased ZrSiO_4_ + SiC particles, single-phased ZrSiO_4_ particles, and single-phased SiC particles [24]. (ZrSiO_4_-SiC)/Al composites based on a combination of 3.75 wt.% ZrSiO_4_ + 11.25 wt.% SiC particles showed the lowest wear rates at various temperatures (50–300 °C) and applied loads (1 kg and 5 kg). There appears to be potential for dual-phased multi-scale ceramic particles to further improve the strength and wear resistance of Al matrix composites. However, studies on the tensile properties and abrasive wear behaviors of in situ dual-phased multi-sized (TiC-TiB_2_)/Al composites have not been conducted.

In the present investigation, we prepared in situ multi-sized (TiC-TiB_2_)/Al-Cu-Mg-Si composites using the methods of combustion reaction synthesis, combustion synthesis, hot pressed sintering and hot extrusion. The effects of in situ multi-sized TiC-TiB_2_ particles on the microstructure, tensile properties, and abrasive wear behaviors of the composites were analyzed. The abrasive wear mechanisms for the Al-Cu-Mg-Si alloys and the (TiC-TiB_2_)/Al-Cu-Mg-Si composites for various applied loads and abrasive particle sizes wereinvestigated.

## 2. Experimental Procedure

**Figure 1:**
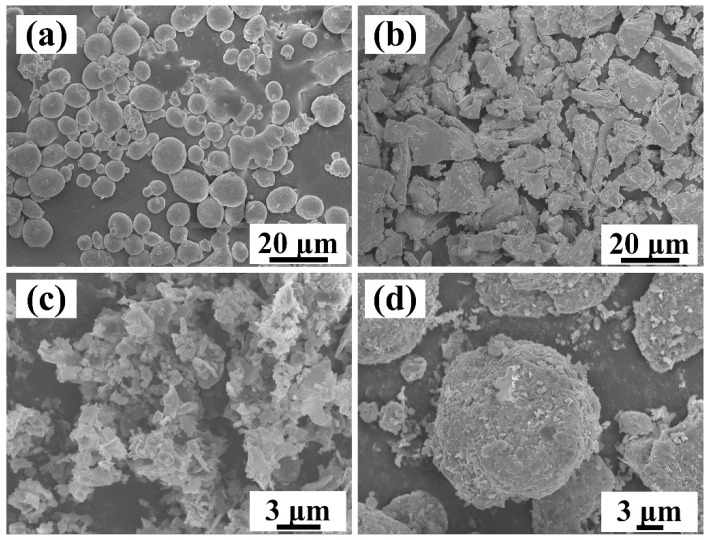
SEM images of (**a**) Al-Cu-Mg-Si alloy powder, (**b**) Ti powder (**c**) B_4_C powder and (**d**) mixed powders of Al, Ti and B_4_C powders after ball-milling.

Al-4.7Cu-0.32Mg-0.44Si alloy powder (~13 µm, 99% purity, Zhe Jiang Bai Nian Yin Industry & Trade Co., Ltd., Jinhua, China), Ti powder (~25 µm, 99.5% purity, Beijing Xingdali Property Management Company, Beijing, China) and B_4_C powder (~1.5 µm, 98% purity, Dunhua Zhengxing Abrasive Co., Ltd., Dunhua, China) were used in this study, as shown in Figure 1a–c. The nominal compositions of the (TiC-TiB_2_)/Al-Cu-Mg-Si composites are presented in Table 1. The molar ratio of Ti:B_4_C was 3:1.

**Figure 2:**
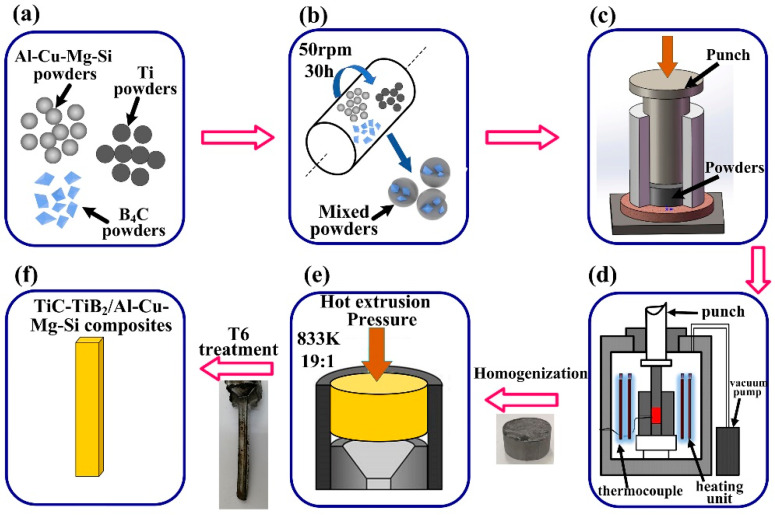
Process flowchart for the preparation of (TiC-TiB_2_)/Al-Cu-Mg-Si composites in Al-Ti-B_4_C system by combustion synthesis and vacuum hot-pressing and assisted by a hot-extrusion process. (**a**) Raw powders, (**b**) ball milling, (**c**) cold pressing, (**d**) combustion synthesis, (**e**) hot extrusion, and (**f**) the prepared (TiC-TiB_2_)/Al-Cu-Mg-Si composites.

Figure 2 shows the process flowchart for the preparation of in situ (TiC-TiB_2_)/Al-Cu-Mg-Si composite. As shown in Figure 2b, the powders were ball-milled for 30 h at 50 rpm. The grinding bodies used were ZrO_2_ balls. A ball-to-powder weight ratio of 50:1 was used. The ball-milling treated powders used can be seen in Figure 1d. The mixed powders were cold-pressed into cylinders (φ45 mm × 30 mm), as shown in Figure 2c. The combustion synthesis process of the cylinder was performed in a vacuum furnace at a heating rate of 30 K/min, as shown in Figure 2d. When the measured temperature suddenly increased, the combustion synthesis reaction started, and the reactant cylinder was rapidly pressed by an axial stress of 50 MPa for 20 s. As the furnace temperature decreased, the sintered (TiC-TiB_2_)/Al-Cu-Mg-Si composites were obtained. When the content of the Ti-B_4_C reactants increased from 10 wt.% to 40 wt.%, the maximum combustion temperature of the reaction system increased from 1580 K to 1800 K. The Al-4.7Cu-0.32Mg-0.44Si matrix alloy used for comparison was prepared using a hot-pressed sintering method (873 K for 2 h). Before hot extrusion, the Al-4.7Cu-0.32Mg-0.44Si matrix alloy and composites were homogenized at 758 K for 12 h. The Al matrix alloy and composites were hot-extruded at 833 K with an extrusion ratio of 19:1, as shown in Figure 2e. The extruded sheets of matrix alloy and composites were subjected to T6 heat treatment, which involved solution treatment (778 K, 2 h), quenching in water, and artificial aging (433 K, 18 h), as shown in Figure 2f.

XRD (D/Max 2500PC, Rigaku, Tokyo, Japan), an SEM (Tescan vega3 XM, Tescan, Brno, Czech Republic) equipped with energy dispersive spectroscopy (EDS) and Oxford NordlysMax EBSD detector, a field emission scanning electron microscope (FESEM, JSM 6700F, JEOL, Tokyo, Japan), and TEM (JEM 2100F, JEOL, Tokyo, Japan) were used for the detailed characterizations. The step size in EBSD characterization was 0.2 µm. The samples were first polished with SiC grinding paper and then electro-polished with 10% HClO_4_-90% ethanol solution at 253 K. About 1000 particles in the FESEM images were randomly selected; the maximum sizes of the particles were used to calculate the size distribution of the prepared TiC-TiB_2_ particles.

The samples used for tensile tests and abrasive wear tests were taken from the Al-4.7Cu-0.32Mg-0.44Si alloy and composite plates along the extrusion direction (ED). Tensile mechanical tests were performed at room temperature using an MTS-810 machine (MTS Systems Corporation, Minneapolis, MN, USA) with a strain rate of 3 × 10^−4^ s^−1^. Dog-bone samples (10 mm in gauge length, 4 mm in width, and 2 mm in thickness) were used for tensile tests. The abrasive wear experiments were carried out on a wheeled abrasion tester (MLH-30, Zhangjiakou Chengxin Test Equipment Manufacturing Co., Ltd., Zhangjiakou, China) at 298 K [27]. The dimensions of the sample used for wear-testing were 30 mm in ED, 5 mm in the transverse direction (TD), and 5 mm in the normal direction (ND), respectively. Applied loads of 5, 15 and 25 N and Al_2_O_3_ abrasive papers with grits of 360 (~40 µm), 600 (~23 µm) and 1000 (~13 µm) were selected to characterize the abrasive wear behaviors of the Al-4.7Cu-0.32Mg-0.44Si alloy and composites. The total sliding distance was 120 m. The volume wear rates (WR) were calculated by dividing the mass loss by the actual density (ρ_actual_) of the samples. The ρ_actual_ was measured by Archimedes’ method. The mass loss of the sample was measured by a high-precision electronic balance (0.0001 g). The micro-hardness tests were performed using a Vickers hardness tester (1600-5122VD Buehler, Feasterville, PA, USA), using an applied load of 5 N and a dwell time of 10 s for 10 times. The abrasive surface roughness (Rt) of samples was measured using a laser scanning confocal microscope (OLYMPUS LEXT OLS3000, Olympus, Tokyo, Japan). The Rt value represents the height difference between the highest point and the lowest points of the worn surface.

## 3. Results and Discussion

**Figure 3:**
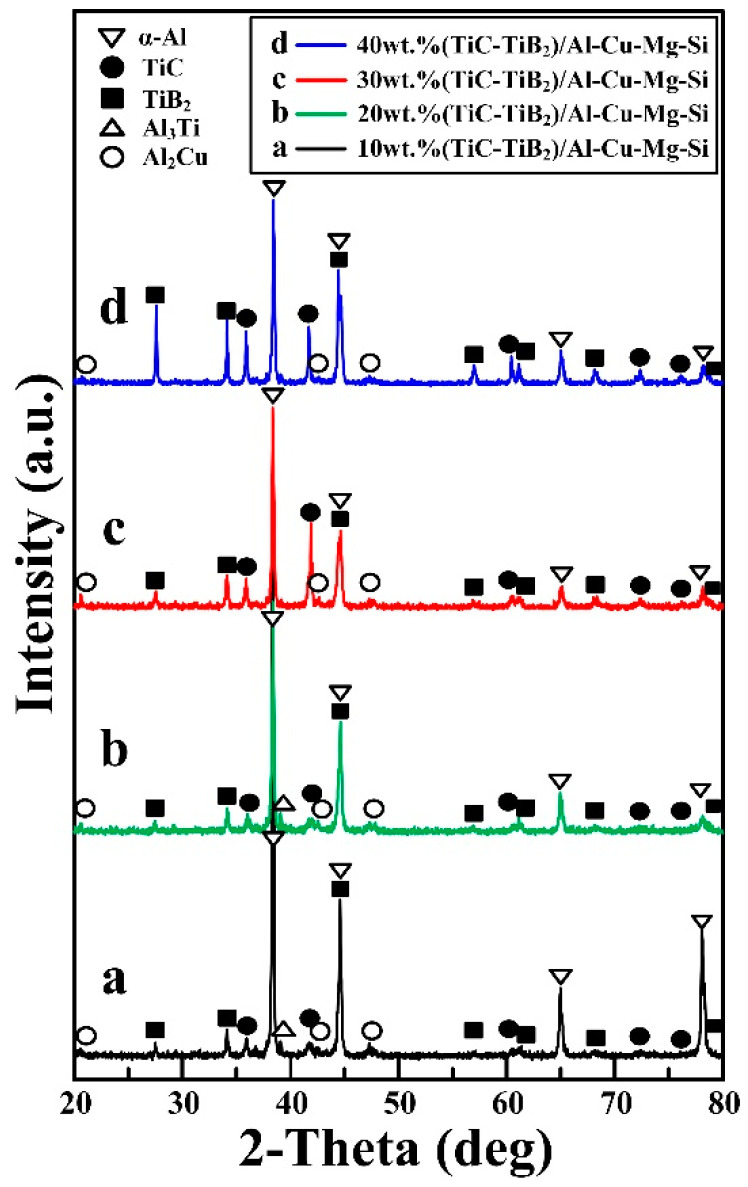
XRD patterns of prepared (TiC-TiB_2_)/Al-Cu-Mg-Si composites with different TiC-TiB_2_ particle content.

Figure 3 shows the XRD analysis results of the prepared in situ (TiC-TiB_2_)/Al-4.7Cu-0.32Mg-0.44Si composites with different TiC and TiB_2_ particle content. The reaction products of the Al-Ti-B_4_C systems mainly consisted of Al, CuAl_2_, TiC and TiB_2_ phases. An intermediate Al_3_Ti phase was observed in the 10 wt.% and 20 wt.% (TiC-TiB_2_)/Al-4.7Cu-0.32Mg-0.44Si composites due to insufficient reaction of the Al-Ti-B_4_C systems. The intensity of the detected TiC and TiB_2_ phases increased with increasing TiC and TiB_2_ particle content from 10 wt.% to 40 wt.%.

**Figure 4:**
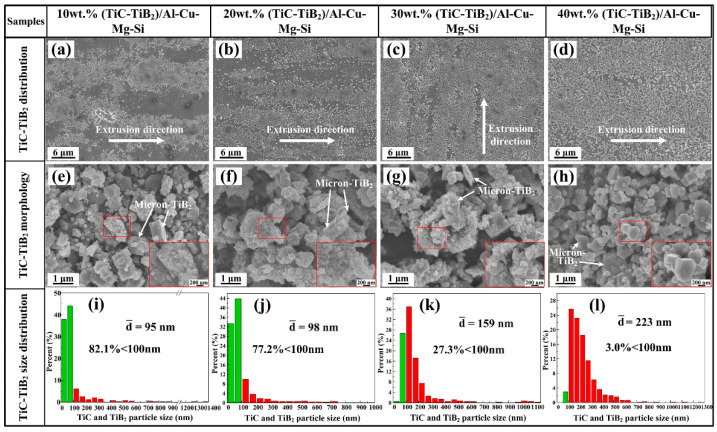
FESEM images of TiC-TiB_2_ particle distribution and morphology, and size distribution diagrams. (**a**,**e**,**i**) 10 wt.% (TiC-TiB_2_)/Al-4.7Cu-0.32Mg-0.44Si; (**b**,**f**,**j**) 20 wt.% (TiC-TiB_2_)/Al-4.7Cu-0.32Mg-0.44Si; (**c**,**g**,**k**) 30 wt.% (TiC-TiB_2_)/Al-4.7Cu-0.32Mg-0.44Si; (**d**,**h**,**l**) 40 wt.% (TiC-TiB_2_)/Al-4.7Cu-0.32Mg-0.44Si. The green bars in Figure 4i–l represent the percentage of nano-sized particles, while the red represents the percentage of sub-micron and micron sized particles.

As shown in Figure 4a–d, the TiC-TiB_2_ particles in (TiC-TiB_2_)/Al-4.7Cu-0.32Mg-0.44Si composites showed a river-like distribution, which improved as the TiC and TiB_2_ particle content increased. The 30 wt.% and 40 wt.% (TiC-TiB_2_)/Al-4.7Cu-0.32Mg-0.44Si composites showed more uniform distribution of TiC and TiB_2_ particles. Figure 4e–l show the morphologies and the size-distribution maps of the extracted TiC and TiB_2_ particles. The synthesized TiC and TiB_2_ particles exhibited a large size span from 20 nm to 1.3 μm. With TiC and TiB_2_ particle content increasing from 10 wt.% to 40 wt.%, the average particle size exhibited obvious increments (from 95 nm to 223 nm), while the percentage of nano-sized TiC-TiB_2_ particles decreased significantly (from 82.1% to 3.0%). In the combustion reaction synthesis process of the Al-Ti-B_4_C system, an Al-Ti liquid phase formed initially, followed by an Al-Ti-B-C liquid phase that formed with the diffusion of [C] and [B] from the B_4_C particles. With continuous decomposition of B_4_C, the [Ti], [B], and [C] reached critical reaction concentrations, resulting in reaction between [Ti]-[B] and [Ti]-[C] with the precipitation of TiC and TiB_2_ particles [28,29,30]. The temperature for the generation of TiC and TiB_2_ particles was the maximum combustion temperature of the reaction system. As the content of the Ti-B_4_C reactants in the Al-Ti-B_4_C system increased from 10 wt.%, to 20 wt.%, to 30 wt.% to 40 wt.%, the maximum combustion temperature of the reaction system increased from 1580 K, to 1642 K, to 1702 K to 1800 K, respectively. Due to the exponential relationship between the crystal growth and the combustion temperature [30], the size of the precipitated TiC-TiB_2_ particles increased with increasing combustion temperature. This caused an increase in Ti-B_4_C reactant content resulting in an increase in the size of the precipitated TiC-TiB_2_ particles. It is of note that the lower diffusion rate of [B] in the molten Al was not favorable for generation of homogenously distributed [B] rich regions [28,29]. The violent reaction between [Ti] and [B] released a large amount of heat, resulting in substantial growth of precipitated TiB_2_ particles. Hexagonally shaped TiB_2_ particles of micron and sub-micron size were identified, as shown in Figure 4e–h.

**Figure 5:**
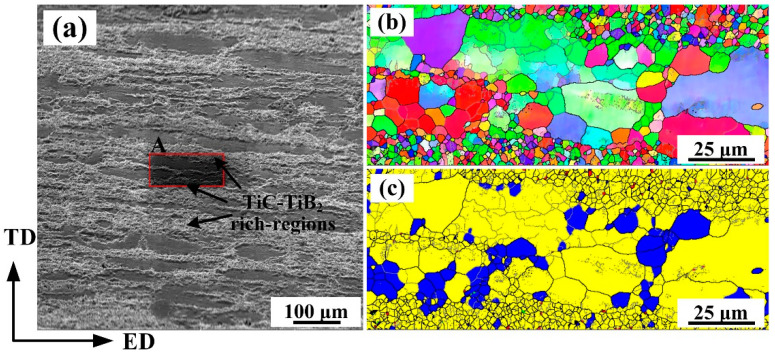
(**a**) SEM image, (**b**) inverse pole figure (IPF) image and (**c**) recrystallized microstructure EBSD image of the extruded 10 wt.% (TiC-TiB_2_)/Al-4.7Cu-0.32Mg-0.44Si composite after T6 heat treatment.

Figure 5a shows an SEM image for the 10 wt.% (TiC-TiB_2_)/Al-4.7Cu-0.32Mg-0.44Si composite after T6 heat treatment. Figure 5b,c show the IPF and recrystallized microstructure maps of area A in Figure 5a. The high-angle grain boundaries (HAGBs, ≥15°) and low-angle grain boundaries (LAGBs, 2–15°) are marked by black lines and gray lines, respectively. Figure 5c shows the deformed grains, sub-grains and recrystallized grains of the composite, which are marked by red, yellow and blue colors, respectively. The composite had a bimodal microstructure containing large-sized sub-grains and fine recrystallized grains. The α-Al grain sizes near the TiC- and TiB_2-_particle-rich regions (0.3 μm) were much smaller than those near the lean regions (34.5 μm). Dynamic recrystallization could not be avoided during hot extrusion. The nano-sized and sub-micro-sized TiC and TiB_2_ particles distributed on the grain boundaries (GBs) may inhibit the rotation and merging of GBs and the growth of α-Al grains [9,30]. Accumulated dislocations in the inner α-Al grains were caused by the TiC and TiB_2_ particles and then LAGBs formed, as seen in Figure 5b. It is possible that multi-sized α-Al grains result in superior mechanical properties of the composites [15].

**Figure 6:**
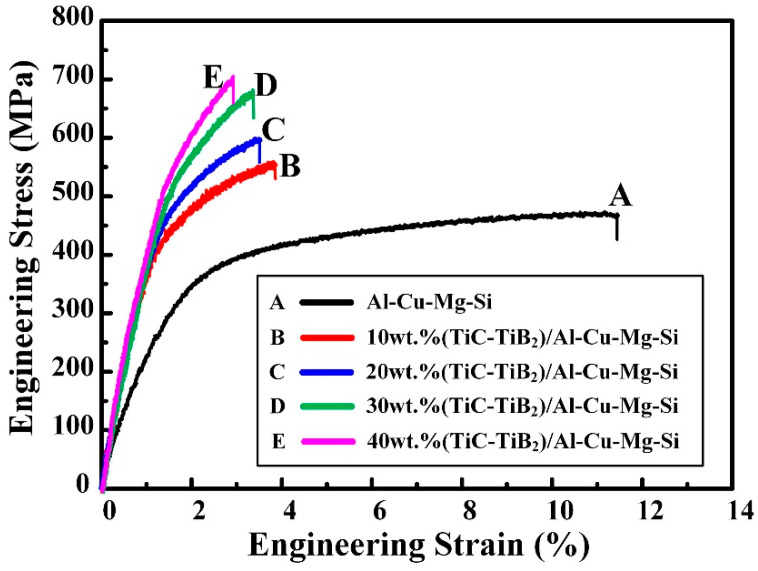
The room-temperature tensile engineering stress-strain curves of the Al-4.7Cu-0.32Mg-0.44Si alloy and in situ (TiC-TiB_2_)/Al-4.7Cu-0.32Mg-0.44Si composites.

The tensile mechanical properties, hardness and actual density of the Al-4.7Cu-0.32Mg-0.44Si alloy and in situ (TiC-TiB_2_)/Al-4.7Cu-0.32Mg-0.44Si composites are presented in Figure 6 and Table 2. The hot-extrusion process effectively consolidated the composites to produce a high relative density (100%). As has been reported previously, the dense composites showed excellent strength and hardness [15,19,31,32,33]. The composites exhibited superior σ_0.2_, σ_UTS_ and HV values to those of the matrix alloy. The σ_0.2_, σ_UTS_ and HV values of the composite increased with increasing TiC-TiB_2_ content. Orowan strengthening effects and grain-boundary pinning effects were the main strengthening mechanisms for the prepared (TiC-TiB_2_)/Al-4.7Cu-0.32Mg-0.44Si composites. As the TiC and TiB_2_ particle content increased and the particle distribution improved, the plastic deformation resistance of the composites increased. The 40 wt.% (TiC-TiB_2_)/Al-4.7Cu-0.32Mg-0.44Si composite showed the highest σ_0.2_ (569 MPa), σ_UTS_ (704 MPa) and HV (286 HV) values, approximately 74%, 51% and 110% higher, respectively, than the corresponding values for the Al-4.7Cu-0.32Mg-0.44Si alloy.

**Figure 7:**
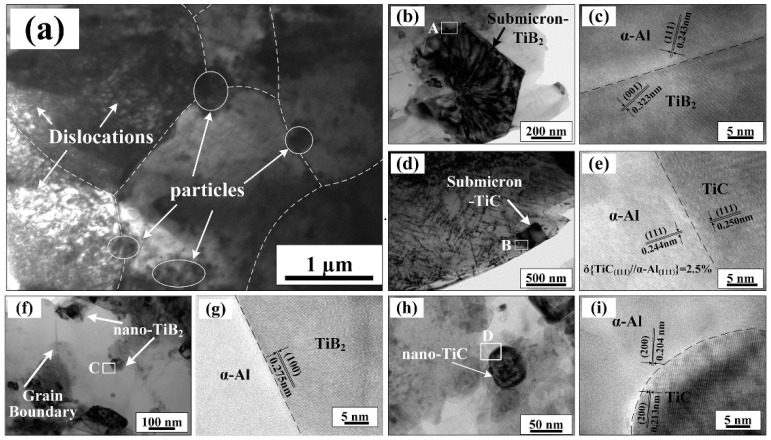
(**a**) TEM microstructure micrograph of in situ 10 wt.% (TiC-TiB_2_)/Al-4.7Cu-0.32Mg-0.44Si composite after room-temperature tensile test. TEM images and corresponding HRTEM of (**b**,**c**) a sub-micron TiB_2_ particle, (**d**,**e**) a sub-micron TiC particle, (**f**,**g**) a nano-TiB_2_ particle, and (**h**,**i**) a nano-TiC particle.

Figure 7a shows a TEM microstructure image of the 10 wt.% (TiC-TiB_2_)/Al-Cu-Mg-Si composite following the hot-extrusion process and heat treatment. Multi-sized TiC and TiB_2_ particles, marked by circles, were located at the inner and grain boundaries (GBs) of α-Al grains. Hexagonally shaped submicron-sized TiB_2_ particles of approximately 800 nm diameter are shown in Figure 7b. Figure 7c shows the corresponding HRTEM image of zone A in Figure 7b. The clean and continuous TiB_2_-Al interface helped to increase the strength of the composites [23,30]. Figure 7d shows an image of a cubic-shaped sub-micron TiC particle of about 300 nm width. An HRTEM image of zone B in Figure 7d is shown in Figure 7e, illustrating a clean TiC-Al interface with mismatched low lattice (2.5%). Figure 7f,g show the TEM image and corresponding nano-sized TiB_2_ particle. Figure 7h,i show nano-sized TiC particles with spherical shapes. Figure 7g,i show the HRTEM images of zone C and zone D in Figure 7f,h, respectively. The TiC and TiB_2_ particles, located in the interior of α-Al grains effectively restricted the dislocation movements, generated dislocation accumulations, and increased the strength (σ_0.2_, σ_UTS_) of the composites. TiC and TiB_2_ particles occurring at the GBs could restrain the grain boundary rotations of the α-Al grains and the plastic deformations of the composites, contributing to increased strength and hardness of the composites.

**Figure 8:**
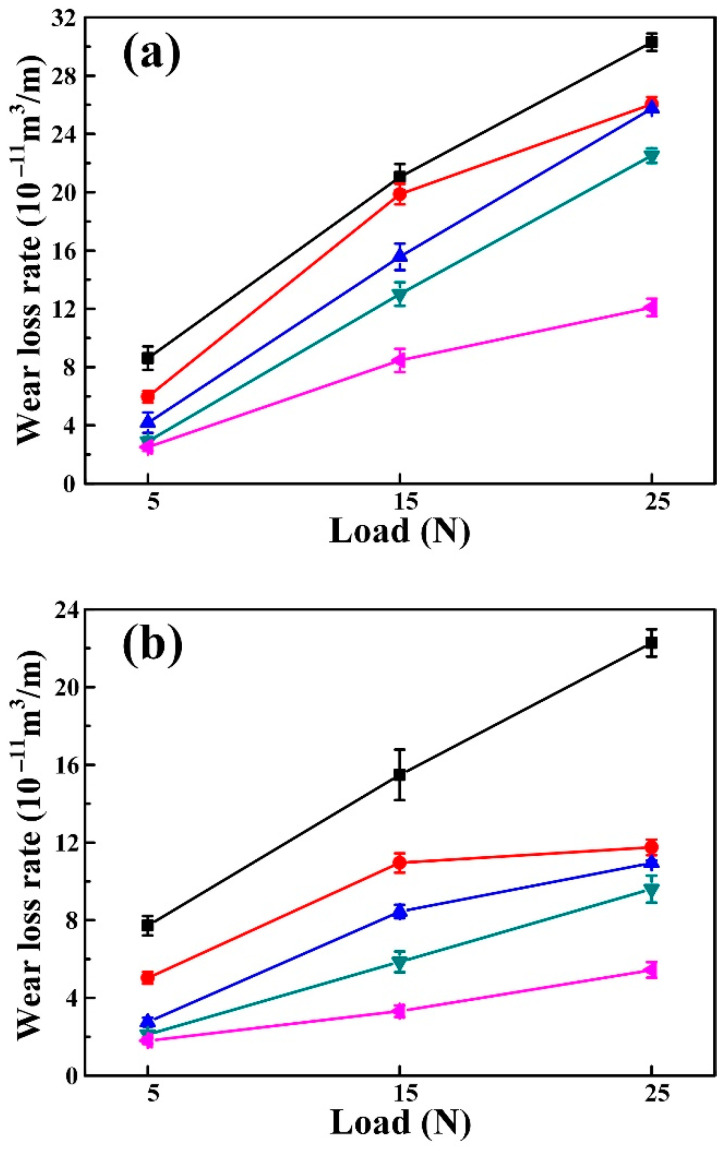

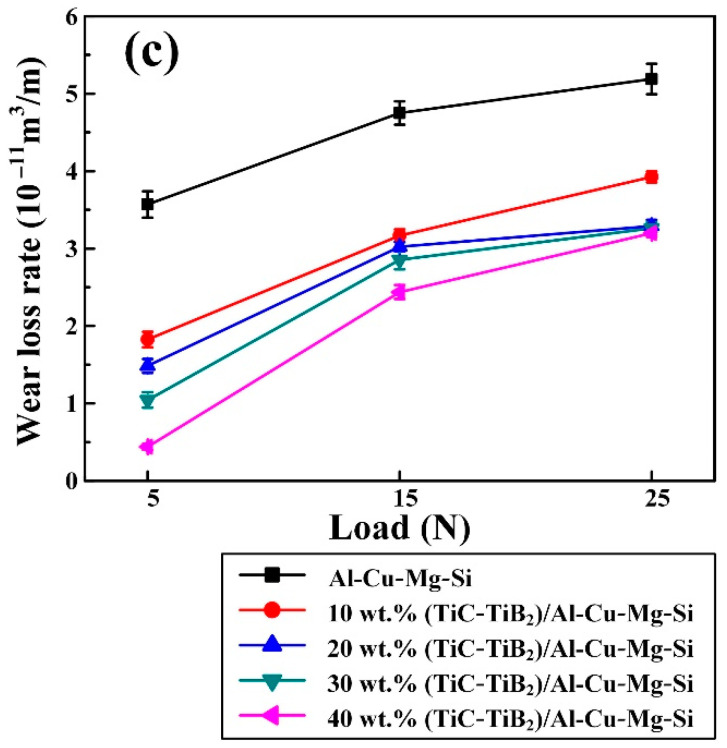
Wear rate vs. applied load under Al_2_O_3_ abrasive size of (**a**) 40 µm, (**b**) 23 µm and (**c**) 13 µm for the Al-4.7Cu-0.32Mg-0.44Si alloy and (TiC-TiB_2_)/Al-4.7Cu-0.32Mg-0.44Si composites.

Figure 8 shows the wear rate (WR, 10^−11^ m^3^/m) of the Al-4.7Cu-0.32Mg-0.44Si alloy and (TiC-TiB_2_)/Al-4.7Cu-0.32Mg-0.44Si composites tested under various loads (5 N, 15 N and 25 N) and Al_2_O_3_ abrasive sizes (13 µm, 23 µm and 40 µm). As observed, the WRs of the Al-4.7Cu-0.32Mg-0.44Si alloy and composites increased almost linearly with increasing applied load. As indicated in Figure 8a, with increasing load from 5 N, 15 N to 25 N, under an Al_2_O_3_ abrasive size of 40 µm, the WRs of the Al-4.7Cu-0.32Mg-0.44Si matrix alloy increased from 8.61, 21.04 to 30.30, while the 40 wt.% (TiC-TiB_2_)/Al-4.7Cu-0.32Mg-0.44Si composite increased from 2.51, 8.46 to 12.09. The relative wear resistance of the 40 wt.% (TiC-TiB_2_)/Al-4.7Cu-0.32Mg-0.44Si composite was increased by 2.43, 1.49, and 1.51 times compared with the matrix alloy, respectively, under loads of 5 N, 15 N, and 25 N, and an abrasive size of 40 µm. The WRs of the matrix alloy and composites decreased with reduction in abrasive particle size. As the Al_2_O_3_ abrasive size decreased from 40 µm, 23 µm to 13 µm under an applied load of 5 N, the WRs of the matrix alloy decreased from 8.61, 7.72 to 3.57, while the 40 wt.% (TiC-TiB_2_)/Al-4.7Cu-0.32Mg-0.44Si composite decreased from 2.51, 1.80 to 0.69. With decreasing Al_2_O_3_ abrasive size from 40 µm, to 23 µm to 13 µm, under a load of 5 N, the relative wear resistance of the 40 wt.% (TiC-TiB_2_)/Al-4.7Cu-0.32Mg-0.44Si composite was 2.43, 3.29, and 4.17 times higher than the Al-4.7Cu-0.32Mg-0.44Si alloy, respectively. Compared with the matrix alloy, the composites showed lower WRs and higher abrasive wear resistance. With increasing TiC and TiB_2_ particle content, the WRs of the composites clearly decreased. The 40 wt.% (TiC-TiB_2_)/Al-4.7Cu-0.32Mg-0.44Si composite showed the lowest wear rates and best abrasive wear resistance at all the tested conditions. The relative abrasive resistance of 40 wt.% (TiC-TiB_2_)/Al-4.7Cu-0.32Mg-0.44Si composite was 4.17 times higher than that of the Al-4.7Cu-0.32Mg-0.44Si alloy under a load of 5 N and an abrasive size of 13 µm.

It is of note that, with increasing load from 5 N, to 15 N to 25 N, with an Al_2_O_3_ abrasive size of 23 µm, the WRs of the 31.9 wt.% (20 vol.%) nano-TiC/Al-4.7Cu-0.32Mg-0.44Si composite increased from 5.84, to 10.73 to 11.21 [34], while the WRs of the 30 wt.% (TiC-TiB_2_)/Al-4.7Cu-0.32Mg-0.44Si composite increased from 2.11, to 5.86 to 9.60. This means that the dual-phased (TiC-TiB_2_)/Al-Cu-Mg-Si composite showed better abrasive wear resistance than the single-phased nano-TiC/Al-Cu-Mg-Si composite.

**Figure 9:**
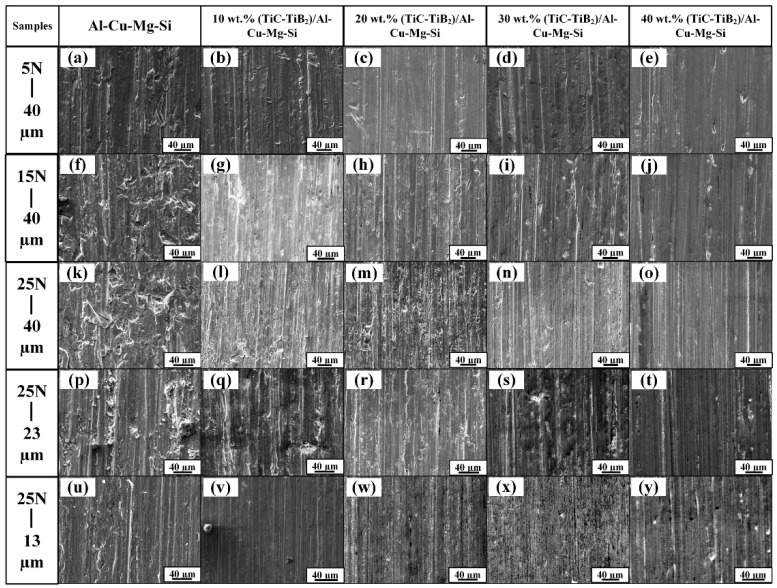
Worn surfaces of Al-4.7Cu-0.32Mg-0.44Si alloy and (TiC-TiB_2_)/Al-4.7Cu-0.32Mg-0.44Si composites tested under various loads and Al_2_O_3_ abrasive sizes. (**a**) Al-4.7Cu-0.32Mg-0.44Si alloy, (**b**) 10 wt.%, (**c**) 20 wt.%, (**d**) 30 wt.%, and (**e**) 40 wt.% (TiC-TiB_2_)/Al-4.7Cu-0.32Mg-0.44Si composites tested under load of 5 N and Al_2_O_3_ abrasive size of 40 μm; (**f**) Al-4.7Cu-0.32Mg-0.44Si alloy, (**g**) 10 wt.%, (**h**) 20 wt.%, (**i**) 30 wt.%, and (**j**) 40 wt.% (TiC-TiB_2_)/Al-4.7Cu-0.32Mg-0.44Si composites tested under load of 15 N and Al_2_O_3_ abrasive size of 40 μm; (**k**) Al-4.7Cu-0.32Mg-0.44Si alloy, (**l**) 10 wt.%, (**m**) 20 wt.%, (**n**) 30 wt.%, and (**o**) 40 wt.% (TiC-TiB_2_)/Al-4.7Cu-0.32Mg-0.44Si composites tested under load of 25 N and Al_2_O_3_ abrasive size of 40 μm; (**p**) Al-4.7Cu-0.32Mg-0.44Si alloy, (**q**) 10 wt.%, (**r**) 20 wt.%, (**s**) 30 wt.%, and (**t**) 40 wt.% (TiC-TiB_2_)/Al-4.7Cu-0.32Mg-0.44Si composites tested under load of 25 N and Al_2_O_3_ abrasive size of 23 μm; (**u**) Al-4.7Cu-0.32Mg-0.44Si alloy, (**v**) 10 wt.%, (**w**) 20 wt.%, (**x**) 30 wt.%, and (**y**) 40 wt.% (TiC-TiB_2_)/Al-4.7Cu-0.32Mg-0.44Si composites tested under load of 25 N and Al_2_O_3_ abrasive size of 13 μm.

In the abrasive wear process, an increase in applied load would contribute to an increase in the contact zone between the specimens and the abrasives [35]. The penetration ability of the Al_2_O_3_ abrasives increased as well as the micro-ploughing efficiency, which caused the increases in the WRs for the matrix alloy and composites. This was confirmed by the worn surface, as shown in Figure 9; with severe plastic deformation, the grooves became deeper and wider in the matrix alloy and the composites when the applied load increased. It can be inferred that micro-ploughing was the main abrasive wear mechanism for the Al-4.7Cu-0.32Mg-0.44Si alloy and the (TiC-TiB_2_)/Al-4.7Cu-0.32Mg-0.44Si composites at an Al_2_O_3_ abrasive size of 40 µm.

**Figure 10:**
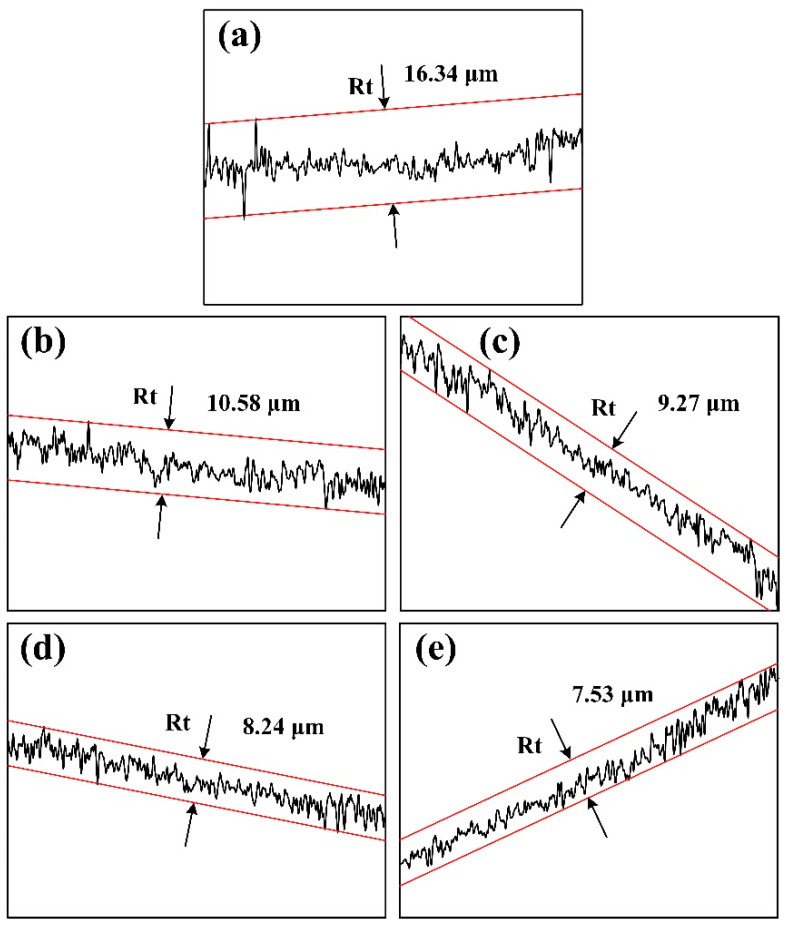
The roughness curves of (**a**) Al-4.7Cu-0.32Mg-0.44Si alloy and (TiC-TiB_2_)/Al-4.7Cu-0.32Mg-0.44Si composites with TiC-TiB_2_ particles content of (**b**) 10 wt.%, (**c**) 20 wt.% (**d**) 30 wt.% and (**e**) 40 wt.% under 5 N load and 13 µm Al_2_O_3_ abrasive size.

As indicated in Figure 9, the worn surface of the composites became smoother with less debris and formed shallower and narrower grooves as TiC-TiB_2_ particle content increased. As shown in Figure 10, with increase in TiC-TiB_2_ particle content, the measured maximum value of Rt decreased from 16.34 μm in the Al matrix alloy, 10.58 μm in the 10 wt.% (TiC-TiB_2_)/Al-4.7Cu-0.32Mg-0.44Si composite, 9.27 μm in the 20 wt.% (TiC-TiB_2_)/Al-4.7Cu-0.32Mg-0.44Si composite, 8.24 μm in the 30 wt.% (TiC-TiB_2_)/Al-4.7Cu-0.32Mg-0.44Si composite, to 7.53 μm in the 40 wt.% (TiC-TiB_2_)/Al-4.7Cu-0.32Mg-0.44Si composite. The hardness of the composites largely determines the penetration ability of the abrasive particles [19]. The TiC and TiB_2_ particles effectively increased the hardness of the composites (from 192 HV to 286 HV), which reduced the penetration ability as well as the micro-ploughing and micro-cutting ability of the Al_2_O_3_ abrasives. The greater hardness of the composites contributed to a reduction in the wear rates and improvement in the abrasive wear resistance. The TiC and TiB_2_ particles showed superior interfacial bonding with the Al matrix and effectively resisted the plastic deformation of the matrix. As discussed above, the distribution of the TiC and TiB_2_ particles improved significantly, which also contributed to resistance to the non-homogenous plastic deformation of the Al matrix. Then, the micro-ploughing efficiency of the Al_2_O_3_ abrasives decreased. The (TiC-TiB_2_)/Al-4.7Cu-0.32Mg-0.44Si composites with higher TiC-TiB_2_ content showed greatly superior abrasive wear resistance and lower wear rates.

As indicated in Figure 9a, micro-cutting and micro-ploughing occurred simultaneously for the Al matrix alloy when tested under a 5 N load using a 13 µm Al_2_O_3_ abrasive. With increase in the abrasive particle size, the micro-ploughing and micro-cutting efficiency of the Al_2_O_3_ abrasive particles increased as well as the WRs of the specimens. When the abrasive size increased from 13 µm, to 23 µm to 40 µm, grooves and ridges with severe plastic deformation appeared, as shown in Figure 9k,p,u. This result indicated an abrasive wear mechanism transition from micro-cutting and micro-ploughing to micro-ploughing for the Al matrix alloy. The WRs increased from 0.69, to 1.80 to 2.51 for the 40 wt.% (TiC-TiB_2_)/Al-4.7Cu-0.32Mg-0.44Si composite with increase in the Al_2_O_3_ abrasive sizes from 13 µm, to 23 µm to 40 µm. The abrasive wear resistance of the composite was 4.17 times higher than for the Al matrix when tested under a 5 N load with 13 µm abrasive. As shown in Figure 9o,t,y, the worn surface of the composites showed increase in the groove width and depth with increase in abrasive particle size. When the tested abrasive sizes increased from 13 µm, to 23 µm to 40 µm under a 5 N load, the abrasive wear mechanism of the 40 wt.% (TiC-TiB_2_)/Al-4.7Cu-0.32Mg-0.44Si composite changed from micro-cutting to micro-ploughing.

**Figure 11:**
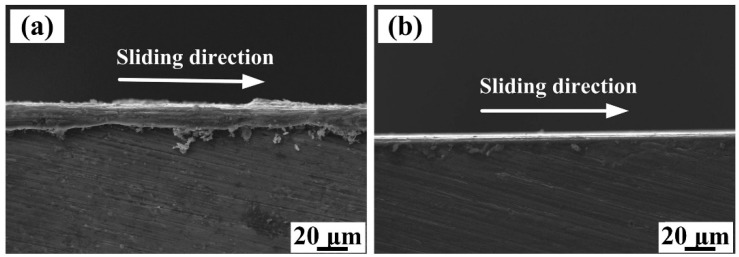
SEM images of longitudinal cross-section worn scar of (**a**) Al-4.7Cu-0.32Mg-0.44Si alloy and (**b**) 40 wt.% (TiC-TiB_2_)/Al-4.7Cu-0.32Mg-0.44Si composite under 5 N load and 13 µm Al_2_O_3_ abrasive size.

Figure 11a,b show cross-sectional SEM images of the worn scar for the Al-4.7Cu-0.32Mg-0.44Si alloy and the 40 wt.% (TiC-TiB_2_)/Al-4.7Cu-0.32Mg-0.44Si composite, respectively, at 5 N load and with 13 µm Al_2_O_3_ abrasive size. Abrasive damage was confined to the subsurface layer of the Al-4.7Cu-0.32Mg-0.44Si alloy and the composite specimens. As shown in Figure 11, the thickness of the cutting layer of the composite was significantly smaller than for the Al alloy.

**Figure 12:**
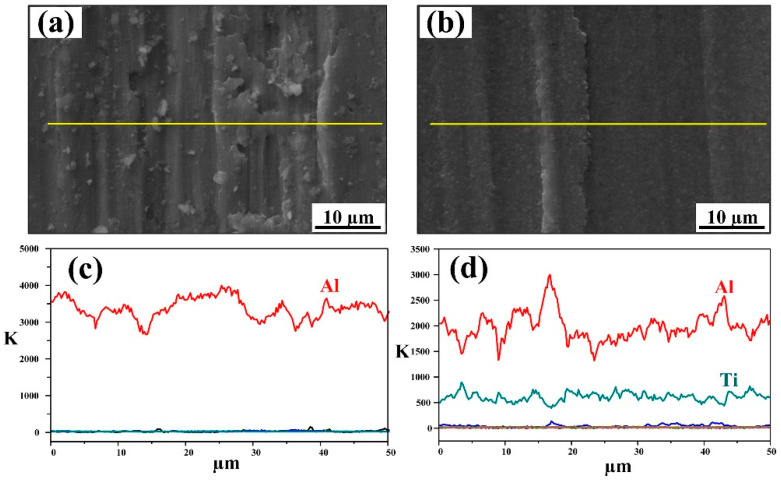
SEM images of worn scar and EDS results of (**a**,**c**) Al-4.7Cu-0.32Mg-0.44Si alloy and (**b**,**d**) 40 wt.% (TiC-TiB_2_)/Al-4.7Cu-0.32Mg-0.44Si composites under 5 N load and 13 µm Al_2_O_3_ abrasive size. (**c**,**d**) show the EDS line scanning analysis results in (**a**,**b**), respectively.

Figure 12 shows higher magnification images of the worn scar and corresponding line EDS results for the Al-4.7Cu-0.32Mg-0.44Si alloy and the 40 wt.% (TiC-TiB_2_)/Al-4.7Cu-0.32Mg-0.44Si composite. As shown in Figure 12a,c, scratches, deep grooves and bridges were observed simultaneously on the worn surface of the Al-4.7Cu-0.32Mg-0.44Si alloy, representing indicators of micro-cutting and micro-ploughing abrasive wear mechanisms. Figure 12d shows the uniform distribution of the Ti element, reflecting the homogenous distribution of TiC and TiB_2_ particles in the composite. The TiC-TiB_2_ particles significantly constrained the plastic deformation of the Al matrix and increased the wear resistance of the composite. The composite presented a much smoother worn surface than the Al alloy, as indicated in Figure 12.

**Figure 13:**
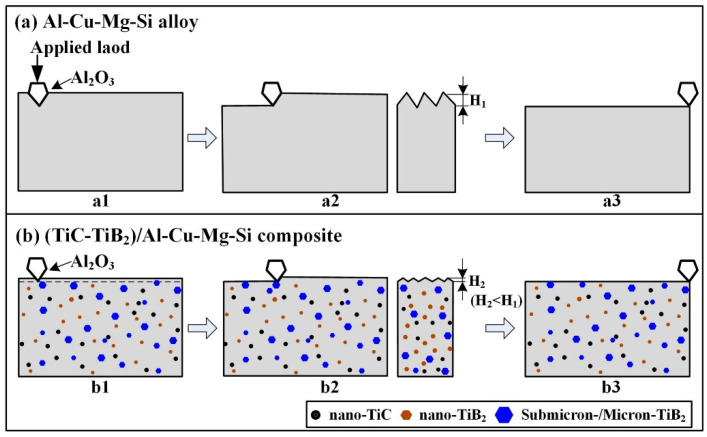
Abrasive-wear behavior schematic view of (**a**) Al-4.7Cu-0.32Mg-0.44Si and (**b**) (TiC-TiB_2_)/Al-4.7Cu-0.32Mg-0.44Si composite.

Figure 13 shows an abrasive wear behavior schematic view of the Al-4.7Cu-0.32Mg-0.44Si alloy and (TiC-TiB_2_)/Al-4.7Cu-0.32Mg-0.44Si composite. In the abrasive wear process for the Al-4.7Cu-0.32Mg-0.44Si alloy, the Al_2_O_3_ abrasives penetrated the Al matrix, moved on the surface and generated the grooves (stage a2), subsequently converting the materials into debris and chips, as shown in Figure 13a. The abrasive wear loss of the Al matrix alloy occurred during the reciprocating movements of the Al_2_O_3_ abrasives (stage a3). Grooves and wear debris formed on the worn surfaces, as shown in Figure 9 and Figure 12. As shown in Figure 13b, the TiC and TiB_2_ particles effectively increased the micro-hardness of the composites, decreased the contact area between the matrix and the Al_2_O_3_ abrasives, and weakened the penetration and micro-cutting efficiency of the Al_2_O_3_ abrasives (stage b2). The TiC and TiB_2_ particles also played a role in the load-bearing elements, which effectively prevented the penetration of the abrasives and then increased the abrasive resistance of the composites [27,36]. Therefore, the composites with higher TiC-TiB_2_ particle content were able to show better abrasive resistance and a lower wear rate. As indicated in Figure 11, the thickness of the plastic deformation zone on the wear surface of (TiC-TiB_2_)/Al-Cu-Mg-Si composite was significantly smaller than that of the Al matrix alloy. Since the penetrating depth and micro-cutting efficiency of the abrasive particles increased with increase in the applied load and abrasive size [37,38], the micro-cutting efficiency of the abrasive particles was improved, leading to an increase in the WRs. When tested with a 13 µm abrasive size and under a 5 N load, the abrasive wear mechanisms were micro-cutting and micro-ploughing for the Al-4.7Cu-0.32Mg-0.44Si alloy, while micro-cutting was the main abrasive wear mechanism for the 40 wt.% (TiC-TiB_2_)/Al-4.7Cu-0.32Mg-0.44Si composite. The composite showed much superior abrasive wear resistance to that of the Al alloy (up to 4.17 times higher). As discussed above, with increase in the applied load (5 N to 25 N) or in the Al_2_O_3_ abrasive size (13 µm to 40 µm), the abrasive wear mechanism of the (TiC-TiB_2_)/Al-4.7Cu-0.32Mg-0.44Si composites changed from micro-cutting and micro-ploughing to micro-ploughing, with formation of much rougher and deeper furrows and much debris. The main abrasive wear mechanisms for the Al-4.7Cu-0.32Mg-0.44Si alloy and the (TiC-TiB_2_)/Al-4.7Cu-0.32Mg-0.44Si composites were micro-ploughing and micro-cutting.

## 4. Conclusions

In situ multi-sized (TiC-TiB_2_)/Al-4.7Cu-0.32Mg-0.44Si composites were synthesized in an Al-Ti-B_4_C reaction system by combining combustion reaction synthesis with hot-pressed sintering and hot extrusion. With increase in TiC-TiB_2_ particle content from 10 wt.% to 40 wt.%, the TiC-TiB_2_ average particle size increased from 95 nm to 223 nm, while the percentage of nano-sized particles reduced from 82.1% to 3.0%. The multi-sized particles effectively improved the tensile properties and abrasive wear resistance of the composites. The mechanical and abrasive wear properties increased with TiC-TiB_2_ particle content. The yield strength, tensile strength, and hardness of the 40 wt.% (TiC-TiB_2_)/Al-4.7Cu-0.32Mg-0.44Si composite (569 MPa, 704 MPa, and 286 HV) were, respectively, 74%, 51%, and 110% higher than those of the matrix alloy (327 MPa, 466 MPa, and 136 HV). The abrasive wear resistance of the 40 wt.% (TiC-TiB_2_)/Al-4.7Cu-0.32Mg-0.44Si composite was 4.17 times greater than for the Al matrix under a 5 N load and with 13 µm Al_2_O_3_ abrasive size. Micro-cutting and micro-ploughing were the main abrasive wear mechanisms for the Al alloy and composites under various applied loads (5 N to 25 N) and for various Al_2_O_3_ abrasive particle sizes (13 µm to 40 µm). The improved abrasive wear performance of the composites was attributed to the excellent bonding of the TiC-Al and TiB_2_-Al interfaces, and improvement in the TiC-TiB_2_ particle distribution.

## Figures and Tables

**Table 1:** Characteristics of the in situ TiC-TiB_2_/Al-Cu-Mg-Si nanocomposites synthesized in Al-Ti-B_4_C systems.

Designed Composition	Used Powders (wt.%)	TiC Content	TiB_2_ Content
10 wt.% (TiC-TiB_2_)/Al-4.7Cu-0.32Mg-0.44Si	90% Al + 7.22% Ti + 2.78% B_4_C	3.01 wt.% (1.78 vol.%)	6.99 wt.% (4.50 vol.%)
20 wt.% (TiC-TiB_2_)/Al-4.7Cu-0.32Mg-0.44Si	80% Al + 14.44% Ti + 5.56% B_4_C	6.02 wt.% (3.71 vol.%)	13.98 wt.% (9.39 vol.%)
30 wt.% (TiC-TiB_2_)/Al-4.7Cu-0.32Mg-0.44Si	70% Al + 21.66% Ti + 8.4% B_4_C	9.03 wt.% (5.81 vol.%)	20.97 wt.% (14.71 vol.%)
40 wt.% (TiC-TiB_2_)/Al-4.7Cu-0.32Mg-0.44Si	60% Al + 28.88% Ti + 11.12% B_4_C	12.05 wt.% (8.12 vol.%)	27.95 wt.% (20.55 vol.%)

**Table 2:** The room-temperature tensile properties, micro-hardness and actual density of the Al-4.7Cu-0.32Mg-0.44Si alloy and in situ (TiC-TiB_2_)/Al-4.7Cu-0.32Mg-0.44Si composites.

TiC-TiB_2_ Content	σ_0.2_/MPa	σ_UTS_/MPa	ε_f_/%	Hardness/HV	Actual Density/g·cm^−3^
Al alloy	$327_{-10}^{+12}$	$466_{-10}^{+16}$	$11.5_{-1.0}^{+2.0}$	136 ± 10	2.796 ± 0.001
10 wt.%	$429_{-11}^{+12}$	$553_{-10}^{+12}$	$3.8_{-0.2}^{+1.6}$	192 ± 8	2.920 ± 0.003
20 wt.%	$483_{-8}^{+11}$	$599_{-10}^{+11}$	$3.5_{-0.1}^{+0.2}$	210 ± 9	3.045 ± 0.002
30 wt.%	$544_{-11}^{+13}$	$680_{-10}^{+13}$	$3.4_{-0.1}^{+0.1}$	268 ± 7	3.192 ± 0.002
40 wt.%	$569_{-10}^{+13}$	$704_{-7}^{+12}$	$2.9_{-0.1}^{+0.3}$	286 ± 5	3.332 ± 0.004

## Data Availability

The data that support the findings of this study are available from the corresponding author upon reasonable request.
